# Research on the Therapeutic Effect and Mechanism of Stir-Roasted Deer Velvet Antler with Ghee on Non-Alcoholic Fatty Liver Disease

**DOI:** 10.3390/nu18030401

**Published:** 2026-01-26

**Authors:** Xuan He, Yinghan Liu, Shuning Cui, Zhenming Yu, Zhongmei He, Ying Zong, Weijia Chen, Jianan Geng, Jia Zhou, Zhuo Li, Yan Zhao, Hongbo Teng

**Affiliations:** 1 Sanjiang Laboratory, Jilin Agricultural University, Changchun 130118, China; 17549668521@163.com (X.H.); liuyh1230633@163.com (Y.L.); cuishuning@jlau.edu.cn (S.C.); hezhongmei@jlau.edu.cn (Z.H.); zongying@jlau.edu.cn (Y.Z.); chenweijia@jlau.edu.cn (W.C.); gengjianan@jlau.edu.cn (J.G.); zhoujia1@jlau.edu.cn (J.Z.); lizhuo@jlau.edu.cn (Z.L.); 2 China State Institute of Pharmaceutical Industry, Shanghai 201203, China; yuzhenming@jlau.edu.cn; 3 International Joint Laboratory for Development of Animal and Plant Resources for Food and Medicine, Changchun 130118, China

**Keywords:** non-alcoholic fatty liver disease, Ghee-Roasted Velvet Antler, traditional chinese medicine

## Abstract

**Objectives**: This study aims to explore the therapeutic effect and mechanism of stir-roasted deer velvet antler with ghee (ZLR) on Non-Alcoholic Fatty Liver Disease (NAFLD). **Methods**: This study used proteomics to analyze the protein composition of roasted deer antler velvet. It established a high-fat diet (HFD)-induced NAFLD rat model and evaluated the therapeutic effects of different dosage groups, including liver injury, oxidative stress, glucose metabolism, steatosis, and insulin homeostasis (via fasting glucose tolerance). Transcriptomics explored the mechanism. Gene expression and Western blot detected lipid metabolism-related gene expression. In vivo experiments validated that ZLR-containing serum alleviates NAFLD and reduces reactive oxygen species levels. **Results**: The results indicated that ZLR could significantly reduce the body weight, liver weight and degree of hepatic steatosis in HFD rats, improve glycolipid metabolism and insulin sensitivity, and alleviate oxidative stress damage. The mechanism involves activating the adenosine monophosphate-activated protein kinase/peroxisome proliferator-activated receptor (AMPK/PPAR) signaling pathway, regulating the expression of lipid metabolism-related genes, promoting fatty acid oxidation, and reducing fat deposition. The results of in vitro experiments show that ZLR-containing serum can effectively reduce lipid droplet production in liver cells and effectively alleviate oxidative stress damage in liver cells. **Conclusions**: The traditional Chinese medicine processed product ZLR can regulate lipid metabolism in the body and alleviate the degree of NAFLD by activating the AMPK and PPAR signaling pathways. It provides new ideas for the clinical treatment of NAFLD.

## 1. Introduction

Recently redefined as metabolic dysfunction-associated fatty liver disease, the condition historically known as non-alcoholic fatty liver disease (NAFLD) is a highly prevalent clinical spectrum of chronic liver disease [1]. Its prevalence continues to rise with improving living standards and the prevalence of sedentary lifestyles, making it a global health concern. The core manifestations of NAFLD encompass hepatic steatosis, dysregulation of lipid and glucose metabolism, and excessive intrahepatic lipid deposition [2]. Ectopic lipid deposition in the liver drives oxidative stress, which in turn perpetuates chronic inflammation [3]. Among these, triglyceride accumulation and lipid droplet formation within hepatocytes constitute a pivotal pathway in the development of liver fibrosis [4], potentially driving NAFLD progression to cirrhosis [5]. Notably, recent studies on neuro-metabolic regulatory networks have confirmed that the neuro-metabolic crosstalk between the central and peripheral nervous systems is one of the core mechanisms mediating the imbalance of hepatic lipid homeostasis—the hypothalamic-autonomic nervous pathway can regulate the hepatic AMP-activated protein kinase (AMPK) signaling axis to affect the key steps of lipid synthesis [6,7], decomposition, and fatty acid oxidation. This mechanism is also supported by research on the systemic metabolic regulation of fatty liver, which confirms a close association between hepatic metabolic disorders and central nervous system dysfunction.

Specifically, hepatic lipid accumulation induces insulin resistance, triggers aberrant glucose and lipid metabolism, and promotes excessive release of pro-inflammatory cytokines, thereby leading to hepatocyte necrosis and apoptosis. Meanwhile, elevated levels of free fatty acid (FFA) not only exacerbate oxidative damage but also further aggravate insulin resistance, facilitating the progression of NAFLD to non-alcoholic steatohepatitis (NASH) and even hepatic fibrosis [8].

In the pathophysiology of NAFLD, energy metabolism disorders constitute the most fundamental pathophysiological basis. Against this backdrop, AMP-activated protein kinase, as a crucial energy and metabolic sensor in the body, plays a regulatory role during energy metabolism imbalances. Dysfunction of this mechanism serves as a key link between nutritional excess and hepatic metabolic abnormalities, contributing to various pathophysiological mechanisms of NAFLD [9]. On the other hand, the dysregulation of lipid homeostasis is a hallmark feature of NAFLD. Peroxisome proliferator-activated receptor alpha (PPARα), a nuclear receptor and master regulator of systemic lipid metabolism, is a key therapeutic target for ameliorating hepatic steatosis and metabolic dysfunction. This is achieved through its potent induction of fatty acid β-oxidation and ketone body production pathways in the liver [10]. Previous studies have demonstrated that multiple signaling pathways, including insulin/PI3K/AKT, cAMP/PKA/CREB, AMPK, and Sirtuin, participate in the regulation of PPARα. Among these, the role of the AMPK pathway is particularly prominent: AMPK activation positively regulates downstream PPARα signaling pathways. On one hand, it suppresses the expression of genes associated with lipogenesis and promotes fat redistribution [11]; on the other hand, by elevating the expression of key regulatory genes at both transcriptional and translational levels, it enhances hepatic fatty acid β-oxidation and concurrently inhibits lipid synthesis, thereby promoting the clearance of lipid deposits [12,13].

Despite continuous advances in modern medicine and deepening understanding of NAFLD pathogenesis, the highly complex and heterogeneous nature of its mechanisms has resulted in a limited number of targeted therapeutic agents being currently available. Therefore, developing drugs capable of effectively intervening in the multifaceted, multi-targeted pathological processes of NAFLD has become an urgent research priority. There is also an urgent need to formulate more targeted direct prevention and treatment strategies.

Deer antler velvet is a precious traditional Chinese medicine, hailed as the “premier animal-derived medicine.” It possesses remarkable therapeutic effects, including strengthening bones and muscles, enhancing immunity, promoting metabolism, alleviating oxidative stress [14], protecting the liver [15], being anti-fatigue [16], and delaying aging [15]. Evidence from the existing literature has documented the hepatoprotective properties of deer antler, with studies highlighting its beneficial roles in managing hepatitis, reducing liver fibrosis, and regulating fatty acid metabolism [14,17]. It is worth noting that Lixin Xu et al. demonstrated that deer antler polypeptides can reduce the degree of liver injury induced by a high-fat diet [18]. The products of enzymatic hydrolysis of deer antler by yuling Ding et al. successfully inhibited the growth of 3T3-L cells and alleviated obesity induced by a high-fat diet in mice [17]. According to the Yushou Yaojie (Compendium of Medicinal Herbs) records, “Deer antler: pungent taste, slightly warm. Deer antler nourishes the kidneys and liver, generates essence and replenishes blood.” Therefore, based on the record in traditional Chinese medicine that “deer velvet antler achieves optimal efficacy when processed with ghee”, this study focuses on stir-roasted deer velvet antler with ghee (ZLR) as a unique TCM resource. It aims to systematically verify its therapeutic efficacy against NAFLD through in vivo and in vitro experiments, clarify its advantages over unprocessed deer velvet antler (LR), further elucidate the molecular mechanism by which it regulates the expression of lipid metabolism-related genes via the AMPK/PPAR signaling pathway, and explore the correlation between the active components enriched after processing and the enhanced efficacy. This study is expected to provide a solid theoretical basis for the high-value development of this resource and offer new candidate strategies and experimental evidence for the clinical prevention and treatment of NAFLD.

## 2. Materials and Methods

### 2.1. Materials and Reagents

Velvet antler of *Cervus canadensis* (batch number: 20240315-JL-KY02-001) was purchased from Jilin Lushengyuan Animal and Plant Sightseeing Park Co., Ltd. Fulinmen anhydrous butter oil was purchased from COFCO Donghai Grain and Oil Co., Ltd.

### 2.2. Preparation of Crispy Fried Deer Antler

Accurately weigh 50 g of processed deer antler velvet and 5 g of refined clarified butter, and place them in a temperature-controllable stir-frying apparatus. Set the temperature at 145 °C and the stirring rate at 50 strokes per minute, then conduct constant-temperature stir-frying for 14 min. Stop the stir-frying process and take the mixture out until the clarified butter is completely adsorbed by the deer antler velvet and its surface presents a uniform oily luster. Allow the stir-fried deer antler velvet to cool naturally to room temperature under ambient conditions, pulverize it using a universal pulverizer, and sift the powder through a 60-mesh standard medicinal sieve. Collect the sieved powder, place it in a dry and clean container, and seal it for subsequent use.

### 2.3. Proteomic Analysis of the Protein Composition in Dried Deer Antler

#### 2.3.1. Protein Extraction and Enzymatic Digestion

Sample lysis and protein extraction were performed using SDT buffer (4% SDS, 100 mM Tris-HCl, 1 mM DTT, pH 7.6). Protein content was quantified using the BCA Protein Quantification Kit (Beyotime Biotechnology, Inc., Nanjing, China). Protein trypsinization followed the filter-aided sample preparation method described by Matthias Mann. Enzymatically cleaved peptides from each sample underwent desalting via C18 column, vacuum centrifugation for concentration, and redissolution in 40 μL of 0.1% formic acid solution for peptide quantification.

#### 2.3.2. Liquid Chromatography–Tandem Mass Spectrometry Analysis

Liquid chromatography–tandem mass spectrometry analysis was performed on a Q Exactive (Thermo Fisher Scientific, Waltham, MA, USA) mass spectrometer coupled with an Easy nLC system (Thermo Fisher Scientific, Waltham, MA, USA), with analysis durations of 60/120/240 min. Peptides were loaded into a reverse-phase capture column in Buffer A (0.1% formic acid), connected to a C18 reverse-phase analytical column. Under IntelliFlow (Entegris, Inc., Billerica, MA, USA) control, linear gradient elution was performed at 300 nL/min using Buffer B (84% acetonitrile and 0.1% formic acid). The mass spectrometer operated in positive ion mode. MS data acquisition employed data-dependent acquisition of the top 10 parent ions, dynamically selecting the most abundant parent ions from the full scan (300–1800 *m*/*z*) for HCD fragmentation. The automatic gain control target was set to 3 × 10^6^, with a maximum injection time of 10 ms. The dynamic exclusion time was 40.0 s. The full scan resolution was set to 70,000 at *m*/*z* 200, while the HCD spectrum resolution was set to 17,500 at *m*/*z* 200 with an isolation width of 2 *m*/*z*. Normalized collision energy was 30 eV, and the underfill rate was defined as 0.1%. The instrument operated with peptide identification mode enabled.

#### 2.3.3. Database Search and Quantification

Raw mass spectrometry data were analyzed using MaxQuant 2.6.7.0 software with its built-in Andromeda database search algorithm. Spectra were searched against the NCBI_Cervus_canadensis_57001_20230423.fasta database and reverse sequences against a contaminant protein database with the following parameters: LC-MS type selected as ‘Standard’ for quantification; specified cystine *N*-acetylcysteine modification as a fixed modification; *N*-deamidation of asparagine and glutamine, *N*-terminal acetylation of proteins, and methionine oxidation as variable modifications, with a maximum of 5 variable modifications allowed; specify trypsin as the digestion enzyme, allowing up to 2 missed cleavage sites; set MS1 primary search mass tolerance to 20 ppm and secondary search to 6 ppm; set MS2 mass tolerance to 20 ppm; and use “Match between runs” for identification transfer. Filter search results at 1% false positive rate at both protein and peptide levels. Proteins annotated as reverse sequences, contaminant proteins, or those identified solely by modification sites were removed. The remaining identified proteins were used for subsequent quantitative analysis.

#### 2.3.4. Bioinformatics Analysis

The classification and functional analysis of differentially expressed proteins were performed using the Gene Ontology (GO) database, the COG (Conserved Ortholog Groups) database, and the KEGG (Kyoto Encyclopedia of Genes and Genomes) database.

### 2.4. Experimental Animals

Thirty 7-week-old male Sprague-Dawley (SD) rats (200–220 g) were purchased from Changchun Yisi Laboratory Animal Technology Co., Ltd. (Changchun, China), with animal license number SYXK (Ji) 2018-0023. The experimental animals were placed in an environment with a room temperature of 20–25 °C, a humidity of 60 ± 5%, and a light and dark cycle for 12 h. They were given a standard diet and distilled water, allowed to eat freely, and had a week to adapt. All animal experiments were carried out in accordance with the “Animal Experiment Guidelines of Jilin Agriculture University” and were approved by the university’s animal ethics committee (Animal Ethics Approval Number: 2023-KJT-021).

### 2.5. Animal Experiment Design

A non-alcoholic fatty liver disease model was induced by feeding rats a high-fat diet (HFD) for 8 weeks. This modeling duration was determined with reference to the previous literature, which is sufficient to induce obvious hepatic steatosis and increase serum liver function indices in rats, consistent with the pathological characteristics of NAFLD. After the 8-week modeling period, the success of model establishment was verified by detecting serum levels of alanine aminotransferase (ALT) and aspartate aminotransferase (AST).

Subsequently, rats with successful modeling were randomly divided into 5 groups using a random number table method, with 5 rats per group, to minimize intergroup bias and ensure baseline consistency in body weight, liver function and metabolic status among all groups. The administration dose was referenced from the Pharmacopoeia of the People’s Republic of China (2020 Edition), and was determined through conversion based on the experimental dry extract yield as well as the body surface area between experimental animals and humans. The groups were designated as follows: model group, Deer Antler (LR) group (1.35 g/kg), stir-roasted deer antler with Ghee high-dose (ZLR-H) group (1.35 g/kg), stir-roasted deer antler with Ghee medium-dose (ZLR-M) group (0.95 g/kg), and stir-roasted deer antler with Ghee low-dose (ZLR-L) group (0.54 g/kg).

For intragastric administration, the medicinal materials were fully mixed with 0.2 mL of 0.1% sodium carboxymethylcellulose (CMC-Na) according to the concentration of each group. The blank group was given an equal volume of 0.1% CMC-Na by intragastric gavage each time, once a day for 4 consecutive weeks.

Throughout the experiment, the administration groups and the model group were continuously fed HFD, while the blank group was fed a normal diet. Individual animals on the HFD were weighed weekly. At the end of the experimental protocol, all rats were anesthetized with sodium pentobarbital before euthanasia was performed.

### 2.6. Histological Staining

Liver tissue samples were fixed with 4% paraformaldehyde (Biosharp, Beijing, China) for 3 days. Hematoxylin and eosin (H&E) staining solution was used to evaluate the morphological changes in liver tissue and lipid droplet formation. For the analysis of white adipose tissue, samples were fixed with a specialized adipose fixative, and the cross-sectional area of adipocytes was measured using ImageJ 1.54r software.

### 2.7. Liver Lipoid O Staining

For Oil Red O staining, liver tissue was cryosectioned under optimal cutting temperature conditions. The resulting sections were first equilibrated to room temperature and subjected to a distilled water rinse. Subsequently, the samples were incubated with freshly prepared 0.5% Oil Red O working solution for 5–15 min. To remove non-specific staining, the sections underwent differentiation in 60% isopropanol followed by a thorough aqueous rinse. Finally, all specimens were permanently sealed with glycerol gelatin mounting medium, preparing them for both qualitative microscopic evaluation and subsequent digital quantification using ImageJ software.

### 2.8. Immunohistochemical (IHC) Analysis

Paraffin sections were subjected to dewaxing, antigen retrieval, blocking treatment, primary antibody incubation, secondary antibody incubation, DAB staining, nuclear counterstaining, dehydration, clearing, mounting, and microscopic examination with photography.

### 2.9. Liver Biochemical Analysis

A series of biochemical assays were performed on liver tissue. Lipid metabolism was profiled by measuring triglyceride (TG) and total cholesterol (TC) levels using kits from Nanjing Jiancheng, alongside FFA content determined with their specific kit (A042-2-1). Simultaneously, oxidative stress status was evaluated by quantifying the levels of malondialdehyde (MDA), superoxide dismutase (SOD) activity, and glutathione (GSH) content with commercial kits obtained (BYabscience, China). All procedures followed the manufacturers’ prescribed guidelines.

### 2.10. Serum Parameter Analysis

ELISA kits were used to detect serum alanine aminotransferase (ALT), aspartate aminotransferase (AST) concentrations, TG, TC, and serum MDA and SOD levels.

### 2.11. Glucose Level Measurement

Following the 4-week ZLR intervention period, we performed systematic metabolic evaluations in the experimental animals. The assessment protocol commenced with an intraperitoneal glucose tolerance test (IPGTT) conducted on rodents subjected to a 16 h fasting period. Animals received a standardized glucose challenge (1 g/kg) through intraperitoneal administration, after which venous blood samples were collected from the tail at predetermined intervals (0, 15, 30, 60, 90, 120, and 150 min) for glucose quantification using a Sinocare monitoring system. After a 72 h washout period, we proceeded with an intraperitoneal insulin tolerance test (IPITT). For this procedure, subjects fasted for 4 h before receiving an insulin bolus (0.2 U/kg) intraperitoneally, with subsequent glycemic measurements obtained following the same temporal sequence and methodological approach.

### 2.12. Rat Liver Transcriptome Sequencing

#### 2.12.1. Experimental Animals and Sample Collection

Rats were euthanized using CO_2_ inhalation. Liver tissue was promptly dissected and separated. Residual blood was rapidly flushed away using pre-chilled 0.9% saline (or PBS). Surface liquid was blotted dry with filter paper. Tissue was then cut into 1–2 mm^3^ pieces. Tissue chunks were immersed in liquid nitrogen for rapid freezing and subsequently transferred to −80 °C for storage.

#### 2.12.2. Total RNA Extraction and Purification

We weighed out 50–100 mg of frozen liver tissue and placed it in a pre-chilled mortar, followed by the addition of liquid nitrogen. The tissue was then ground thoroughly into a fine powder. This tissue powder was transferred into a centrifuge tube pre-loaded with 1 mL of TRIzol reagent and incubated at room temperature for 5 min to ensure the complete lysis of nucleic acid-protein complexes. Subsequently, 200 μL of chloroform was added to the tube, which was then vortexed vigorously for 15 s and allowed to stand at room temperature for an additional 3 min. Upon completion of the standing period, the centrifuge tube was subjected to centrifugation at 12,000× *g* and 4 °C for 15 min, leading to the spontaneous separation of the solution into three distinct layers. A 500 μL aliquot of the upper aqueous phase was carefully aspirated and transferred to a new RNase-free centrifuge tube. An equal volume of isopropanol was added, and the mixture was mixed gently by inversion before being incubated at −20 °C for 30 min to facilitate RNA precipitation.

After incubation, the mixture was centrifuged again at 12,000× *g* and 4 °C for 10 min. The supernatant was discarded, revealing a visible white RNA pellet at the bottom of the tube. The pellet was washed with 1 mL of RNase-free 75% ethanol, followed by centrifugation at 7500× *g* and 4 °C for 5 min. The supernatant was discarded once more, and the centrifuge tube was inverted and air-dried at room temperature for approximately 5–10 min. Careful attention was paid to avoid excessive drying of the pellet, as this could result in RNA insolubility. Finally, the dried pellet was resuspended in 30–50 μL of RNase-free water and incubated at 55–60 °C for 10 min to enhance RNA dissolution. After a brief centrifugation step, the resulting sample was stored at −80 °C for future use.

#### 2.12.3. Transcriptome Sequencing Library Preparation

1 μg of total RNA per sample was used as input for RNA sample preparation to generate sequencing libraries, with index codes added to the attribute sequences of each sample. PCR was performed using Phusion High Fidelity DNA Polymerase, universal PCR primers, and index (X) primers.

#### 2.12.4. Clustering and Sequencing

Indexed samples were clustered using the TruSeq PE Cluster Kit v4-cBot-HS (Illumina) on a cBot clustering generation system according to manufacturer instructions. Following clustering, libraries were sequenced on an Illumina platform to generate paired-end reads.

#### 2.12.5. Differential Expression Analysis

Differential expression analysis between the two groups was performed using edgeR 3.44.0 software. The Benjamini–Hochberg method was applied to control the false discovery rate, thereby adjusting the generated *p*-values. Genes identified by edgeR with adjusted *p*-values < 0.05 were designated as differentially expressed genes (DEGs) and plotted on a volcano plot.

### 2.13. Western Blot

Tissue and cell samples were homogenized in ice-cold RIPA lysis buffer supplemented with protease and phosphatase inhibitors. The resulting lysates were resolved by 8–12% SDS-PAGE and electrophoretically transferred to PVDF membranes (IPVH00010; Merck Millipore, Billerica, MA, USA). Following a blocking step with 5% skim milk, the membranes were probed with specific primary antibodies overnight at 4 °C. Subsequently, they were incubated with a corresponding secondary antibody for 2 h at room temperature. Protein bands were finally visualized using an ECL chemiluminescent substrate (BL520A; Biosharp, China) and imaged. The antibodies used are shown in Table 1.

### 2.14. RT-PCR

Total RNA was extracted from both cellular and tissue samples using TRIzol reagent (RP40002; Biotek Instruments, Inc., Winooski, VT, USA) and subsequently transcribed into complementary DNA with a reverse transcription master mix (R222-01; Vazyme Biotech, Nanjing, China). For quantification of target genes, real-time quantitative PCR was performed with ChamQ SYBR master mix (Vazyme, Q311-02). Relative expression values were determined through the 2^(−ΔΔCT)^ calculation method, normalized against control samples. All oligonucleotide sequences utilized in this study are documented in Table 2.

### 2.15. Preparation of Drug-Containing Serum from Animals

Eight healthy male Sprague-Dawley (SD) rats were selected and subjected to a 3-day acclimatization period. Subsequently, they were randomly divided into three groups based on body weight: the blank group, the velvet antler (LR) administration group, and the stir-fried velvet antler (ZLR) administration group. Rats in the LR group and ZLR group were intragastrically administered with velvet antler powder suspension and Stir-fried velvet antler powder suspension at a dose of 1.35 g/kg daily, respectively, while the blank group received an equal volume of 0.1% sodium carboxymethylcellulose (CMC-Na) via intragastric gavage. The administration was continued for 7 consecutive days. One hour after the final dose, rats were anesthetized with 2% sodium pentobarbital via intraperitoneal injection. We collected blood via the abdominal aorta and separated serum by centrifugation (14,000 rpm, 15 min, 4 °C). We then pooled, filter-sterilized (0.22 μm), and aliquoted the serum from each group before storing it at -80 °C. For in vitro interventions, we diluted the medicated serum in cell culture medium at a predetermined ratio before applying it to cells.

### 2.16. Cell Culture and Treatment

Cell culture and processing of AML12 cells were obtained from Wuhan Ponsure Life Science and Technology Co., Ltd. Cells were cultured in Dulbecco’s modified Eagle medium (DMEM/F-12; Solarbio, Beijing, China) supplemented with 10% fetal bovine serum (FB25015; CLARK BIOSCIENCE, Shanghai, China) and 1% penicillin–streptomycin (T1300). The cultures were maintained at 37 °C in a humidified incubator with 5% CO_2_. Cells were subjected to serum starvation for 12 h in serum-free DMEM/F12, and then incubated again for 24 h with 0.3 mM oleic acid (OA) (01023294; Adamas-Beta, Shanghai, China) in the absence or presence of drug-containing serum from the high-dose administration group.

### 2.17. Sodium Oleate Induction Analysis

Following the seeding of cells into 96-well culture plates, the cells were co-incubated with sodium oleate at various concentrations for 24 h after complete cell adherence was achieved. Subsequently, methyl thiazolyl tetrazolium (MTT) was added to each well, and the cells were incubated successively at 37 °C for 4 h. To fully dissolve the formazan crystals produced during the reaction, dimethyl sulfoxide (DMSO) was introduced, and the absorbance at a wavelength of 490 nm was determined using a microplate reader (Synergy H1, BioTek, Winooski, VT, USA).

### 2.18. Cell Oil Red O Staining

Oil Red O staining solution was prepared in advance in accordance with the kit instructions, allowed to stand for 10 min, and then filtered for subsequent use. Upon completion of cell incubation, the culture medium was aspirated, and the cells were washed 2–3 times with phosphate-buffered saline (PBS). The cells were then fixed with Oil Red O fixative for 20–30 min; after discarding the fixative, they were washed another 2–3 times with distilled water and immersed in 60% isopropyl alcohol for 5 min. Following the removal of isopropyl alcohol, the cells were stained with Oil Red O staining solution for 10–20 min. The staining solution was discarded, and the cells were washed thoroughly with distilled water until no residual excess staining solution was detected. The cells were subsequently incubated in Oil Red O buffer solution for 2–3 min, after which the buffer was discarded. Distilled water was added to cover the cells, and the samples were observed and imaged under a light microscope.

### 2.19. Determination of Intracellular Reactive Oxygen Species

Following the manufacturer’s protocol provided with the kit, the 2′,7′-dichlorodihydrofluorescein diacetate (DCFH-DA) probe was appropriately diluted with serum-free cell culture medium prior to the assay. Upon completion of cell incubation, the original culture medium was carefully aspirated, and the adherent cells were rinsed 2–3 times with phosphate-buffered saline (PBS). Subsequently, an appropriate volume of the pre-diluted DCFH-DA probe working solution was added to the cell culture system. The cells were then incubated in a 37 °C cell incubator for 20 min, after which they were rinsed another 2–3 times with serum-free cell culture medium to thoroughly eliminate the uninternalized probe. Finally, fluorescence signals were visualized and imaged using an inverted fluorescence microscope at an excitation wavelength of 488 nm.

### 2.20. AMP-Activated Protein Kinase Knockout Related Verification

AML12 cells in the logarithmic growth phase were harvested, and the cell density was adjusted to the optimal seeding concentration prior to being seeded into culture plates. Upon reaching a cell confluence of 60–70%, RNA interference (RNAi) assays were performed using small interfering RNA targeting AMP-activated protein kinase. (sequence: 5’-GCAGAGTATGTAGAGCAA-3′ and siNC were synthesized by RiboBio in Guangzhou, China. siAMPK or siNC with a final concentration of 50 nM was transfected into cells using transfection reagents. One hour after transfection, it was changed to complete medium and continued to be cultured for 48 h to verify the silencing effect. Subsequently, the verification method was repeated to culture the cells. After 24 h, the medium with OA was replaced and the culture continued. After 36 h, the TG content of the supernatant was detected, and the cells were stained with oil red O. Finally, protein samples were collected for Western blot detection.

### 2.21. Statistical Analysis

Data are presented as mean ± SD. All statistical analyses and graphing were performed using GraphPad Prism 8.0. Group comparisons were conducted using one-way ANOVA followed by Dunnett’s post hoc test, while differences between two groups were assessed by Student’s *t*-test. Statistical significance is indicated as. * *p* < 0.05, ** *p* < 0.01, *** *p* < 0.001, ^#^ *p* < 0.05, and ^##^ *p* < 0.01. (* indicates the comparison between the drug-treated group and the model group; # indicates the comparison between the model group and the blank control group).

## 3. Results

### 3.1. Determination of Proteins in Roasted Deer Antler Using Quantitative Proteomics

**Figure 1:**
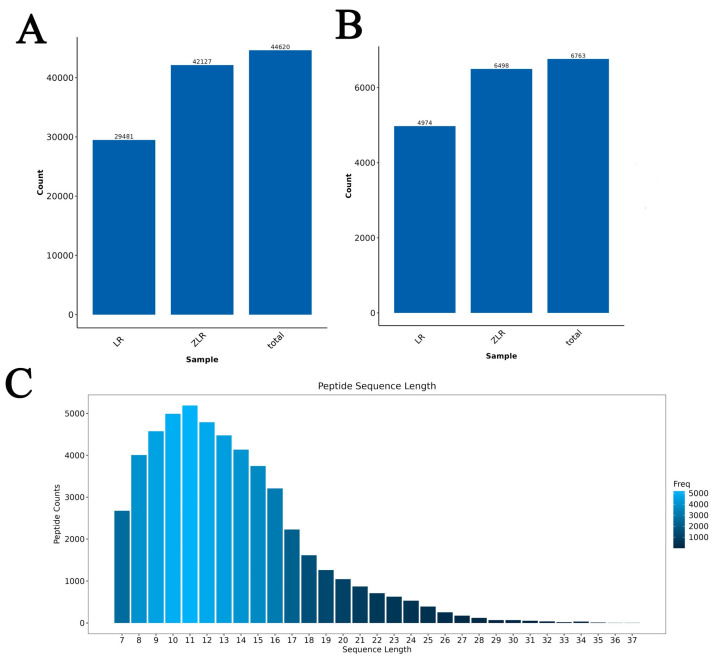
Proteomics Analysis of roasted deer antler velvet. (**A**) Quantification of peptide identification results. (**B**) Quantification of protein identification results. The horizontal axis represents groupings, while the vertical axis denotes the corresponding peptide/protein counts. (**C**) Peptide length distribution. The horizontal axis indicates peptide length, the vertical axis shows peptide counts, and color intensity reflects frequency distribution differences across intervals.

The identification results are shown in Figure 1A,B. Based on a false discovery rate (FDR) threshold of ≤0.01, the LR group identified 29,481 peptides, while the ZLR group identified 42,127 peptides. After removing duplicates, a total of 44,620 peptides were obtained. The LR group identified 4974 proteins, and the ZLR group identified 6498 proteins. After removing duplicates, a total of 6763 proteins were obtained. Most peptides ranged from 7 to 20 amino acids in length (Figure 1C), consistent with the general pattern observed in enzyme digestion and mass spectrometry fragmentation.

### 3.2. Identification and Analysis of Differentially Expressed Proteins (DEP)

**Figure 2:**
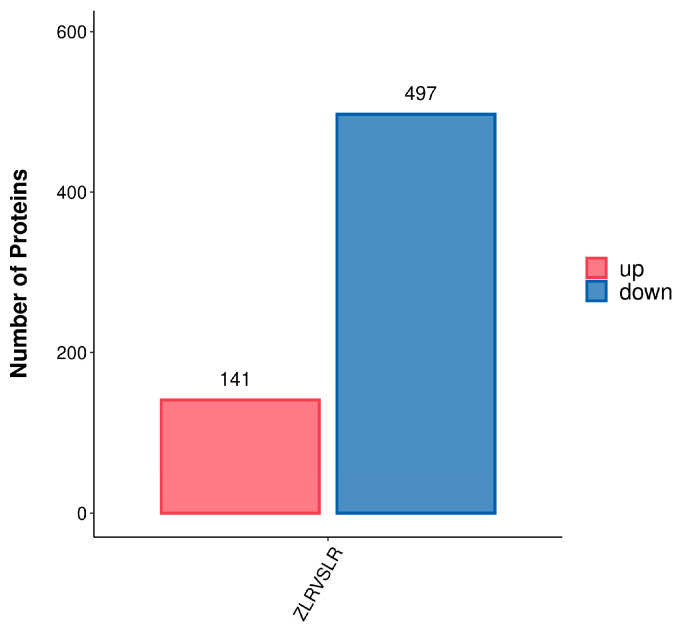
Statistical Chart of Difference Analysis Results.

Compared with LR, ZLR has a total of 647 differentially expressed proteins, among which 141 are up-regulated and 489 are downregulated (Figure 2). The threshold for significant upregulation is based on a change in the expression of differentially expressed proteins greater than 1.5 and less than 1/1.5. Compared with the LR group, ZLR has 1789 unique proteins. Table S1 shows the top ten unique polypeptides in the ZLR group after ghee removal, and Table S2 shows the top ten unique proteins in the ZLR group after ghee removal.

### 3.3. GO Enrichment Analysis and KEGG Enrichment Analysis of DEPs

**Figure 3:**
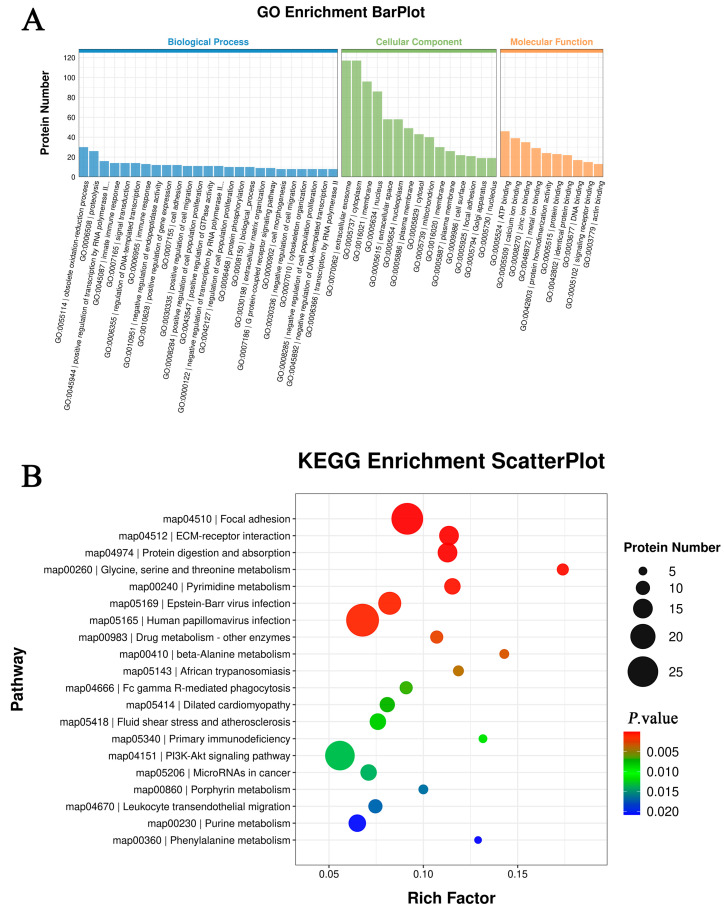
Enrichment analysis results for differentially expressed proteins. (**A**) GO enrichment analysis results. The horizontal axis represents different functional categories or metabolic pathways, while the vertical axis indicates the number of differentially expressed proteins for each GO term. (**B**) KEGG enrichment analysis bubble chart. The horizontal axis displays enrichment factors, and the vertical axis shows KEGG pathway names.

Based on GO enrichment analysis results (biological processes, cellular components, and molecular functions), differentially expressed proteins were grouped into three categories (Figure 3A). GO analysis revealed that DEPs participated in diverse biological processes, including metabolic regulation, gene expression, and immune responses. At the molecular level, DEPs were involved in energy metabolism, substance binding, and genetic information regulation. Among cellular components, GO terms for DEPs encompassed extracellular secretory structures, cytoplasmic metabolic domains, and transmembrane structures, contributing to spatial functions such as secretion, metabolism, and signal transduction.

The KEGG database was utilized to map all differentially expressed proteins. The top 20 KEGG pathways for ZLR and LR are shown in Figure 3B, encompassing metabolic pathways, energy metabolism, prion disease, immune disease pathways-multiple causes, lipid biosynthesis, glucose metabolism/gluconeogenesis regulation, rheumatoid arthritis, cytokine receptor interactions, JAK-STAT signaling, Lewy body dementia, adhesion junctions, multiple sclerosis, hepatitis B virus infection, coagulation and fibrinolytic cascade, Notch signaling pathway, spinal muscular atrophy, Shigella infection, influenza virus infection-type A, viral tumorigenesis, and protein folding and degradation.

### 3.4. Stir-Roasted Deer Antler Improves Liver Steatosis in Rats Fed an HFD

**Figure 4:**
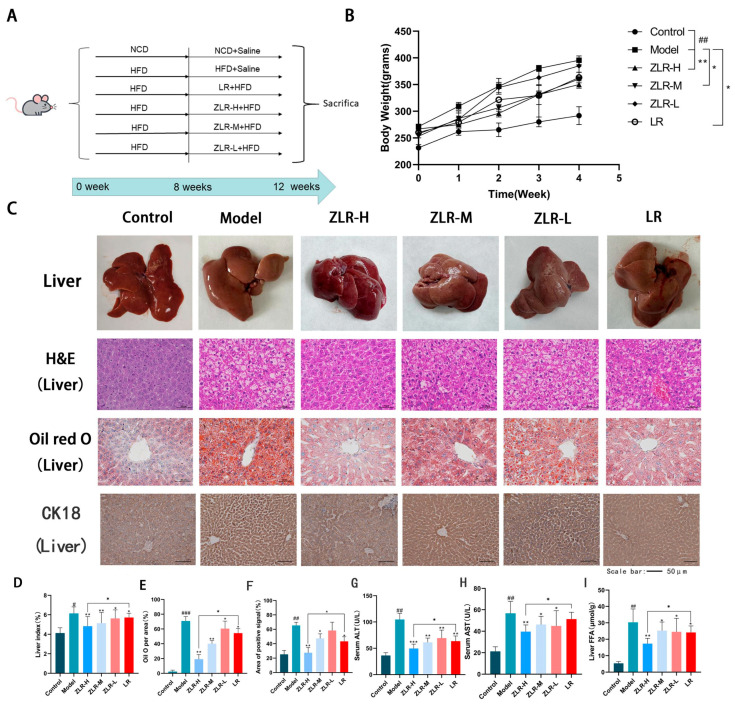
Stir-roasted deer antler can improve the weight and liver fat accumulation induced by HFD. (**A**) Experimental flowchart. (**B**) Weight changes in different groups. (**C**) Liver changes in each group, H&E staining, oil red O, and CK18 staining graphs. (**D**) Quantitative analysis of Liver Indices in rats. (**E**) Oil red O quantification chart. (**F**) Quantification of CK18 signal level. (**G**) AST levels in the serum of rats in each group. (**H**) ALT levels in the serum of rats in each group. (**I**) FFA levels in the liver. The data is shown as the average value ± SD. The n of all groups is 5. * *p* < 0.05, ** *p* < 0.01, *** *p* < 0.001, ^#^ *p* < 0.05, and ^##^ *p* < 0.01, ^###^ *p* < 0.001.(* indicates the comparison between the drug-treated group and the model group; # indicates the comparison between the model group and the blank control group).

To determine the efficacy of stir-roasted deer antler on NAFLD, the process of the HFD-induced SD test is shown in Figure 4A: To evaluate the NAFLD model constructed in mice, the rats first followed a Normal Chow Diet (NCD) and HFD for 8 weeks. After the successful modeling was determined by measuring the AST and ALT of the rats (the results are shown in Table 3).

Subsequently, they were divided into the model group, the deer antler group, the stir-roasted deer antler group (high), the stir-roasted deer antler group (medium), and the stir-roasted deer antler group (low). The treatment group was treated with HFD combined with different doses of stir-roasted deer antler (1.35, 0.95, 0.54 g/kg) and deer antler (1.35 g/kg) for 4 weeks. The model group was fed with HFD and given the same amount of normal saline by gavage for the same period of time. The blank group was fed with the Normal Control Diet (NCD) and intragastric administration of the same amount of normal saline. The research results are shown in Figure 4C. The weight gain curves of the six groups are significantly different. Starting from the 8th week, the weight of the HFD group increased significantly compared with the NCD group. The average weight of each group administered the drug decreased to varying degrees. Notably, the weight gain in the ZLR group was the least significant. Compared with the LR group, the rats in the ZLR group were thinner and had a lower weight (Figure 4B). The livers of rats fed with HFD all showed an overall increase in volume and weight, and morphological abnormalities such as yellowing on the surface. After treatment with ZLR, the liver surface became significantly rosy and the weight decreased, which could significantly alleviate the morphological abnormalities caused by HFD.

The degree of NAFLD was evaluated by measuring the levels of ALT and AST in serum [19], and the results are shown in Figure 4F,G. After administration of high-dose flaky antler or antler, the values of ALT and AST in serum decreased to a certain extent. The levels of FFA, as the main source of NAFLD, decreased significantly after administration in each group (Figure 4H). Among them, the therapeutic effect of the ZLR group was the most obvious. To further evaluate the effect of ZLR on liver steatosis and lipid accumulation in NAFLD rats, H&E staining was used to assess liver tissue steatosis, and oil red O was used to measure the accumulation of liver lipid droplets (Figure 4C). The H&E staining results showed that after 4 weeks of treatment, both the ZLR group and the LR group could alleviate liver injury and hepatic steatosis in NAFLD rats. The results of the oil red O determination showed that both ZLR and LR treatments could improve lipid accumulation in the liver and reduce the formation of lipid droplets in the liver, proving that the administration of both could effectively alleviate liver fat accumulation. And the effect of ZLR is better than that of LR. Subsequently, we stained CK18 in the liver. In the model group, the yellow signal was significantly increased. After drug administration treatment, the yellow signal was significantly reduced, further proving that the symptoms of NAFLD would be improved after ZLR treatment.

### 3.5. Stir-Roasted Deer Antler Reduces Insulin Resistance and Prevents Lipid Accumulation Induced by HFD

**Figure 5:**
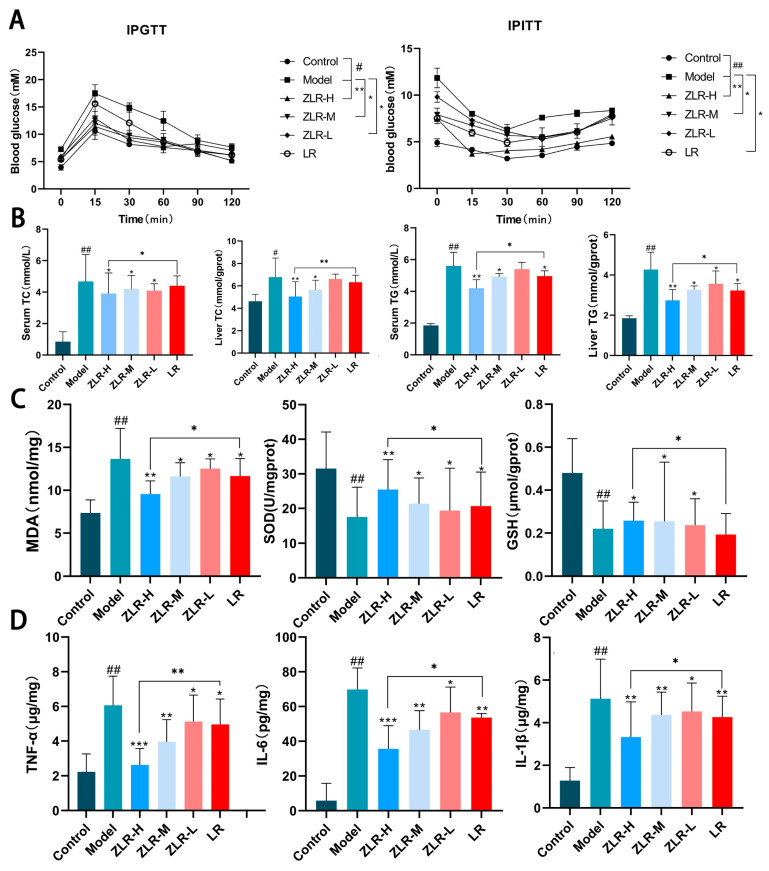
Stir-roasted deer antler improves glucose metabolism, lipid levels, oxidative stress injury of liver cells, and inflammatory factor levels in NAFLD rats. (**A**) Rat IPGTT detection and IPITT detection. (**B**) Levels of TC and TG in the liver and serum. (**C**) The levels of MDA, SOD, and GSH in rat serum. (**D**) Levels of inflammatory factors in serum. The data is shown as the average value ± SD. The n of all groups is 5. * *p* < 0.05, ** *p* < 0.01, *** *p* < 0.001, ^#^ *p* < 0.05, and ^##^ *p* < 0.01. (* indicates the comparison between the drug-treated group and the model group; # indicates the comparison between the model group and the blank control group).

The principal comorbidities of NAFLD encompass hyperlipidemia, obesity, and insulin resistance [20]. The experiment further examined various indicators of NAFLD rats as well as their glucose tolerance and insulin tolerance (IPITT). The results are shown in Figure 5A. Both LR and ZLR treatments can improve insulin resistance in NAFLD rats. Moreover, both have certain effects in improving blood glucose levels. Among them, ZLR can more effectively improve the high fasting blood glucose level induced by HFD. To deeply evaluate the effects of high-dose ZLR on lipid and glucose metabolism in NAFLD rats, the levels of TC and TG in rat serum and liver were detected. As shown in the results (Figure 5B), HFD feeding significantly increased the levels of TC and TG in the serum and liver, while treatment with ZLR and LR could reduce the levels of TC and TG in the serum and liver, and ZLR had a better effect on reducing TG and TC. In conclusion, these results indicate that ZLR can improve the systemic lipid and glucose metabolism in NAFLD rats.

### 3.6. Stir-Roasted Deer Antler Inhibits HFD-Induced Oxidative Stress and Inflammatory Levels in Hepatocytes

Due to the fact that a large amount of FFA can induce the massive generation of reactive oxygen species and trigger subsequent oxidative stress, the values of MDA, SOD, and GSH in the liver change significantly [21]. A significant increase in MDA and decrease in SOD and GSH were observed in the HFD group compared to NCD controls. All treatment groups attenuated these changes, with the ZLR-H group showing superior efficacy in robustly attenuating MDA elevation, enhancing SOD activity, and restoring GSH level (Figure 5C). In the study of NAFLD, evaluating the levels of inflammatory factors can clarify the progression of the disease from NAFLD to the stage of non-alcoholic steatohepatitis [22]. Therefore, we also investigated the impact of stir-roasted deer antler on the changes in the levels of pro-inflammatory factors in the liver. The results showed that, compared with the rats fed with NCD, the levels of TNF-α, IL-6, and IL-1β in the serum of the rats fed with HFD were significantly increased. After 4 weeks of administration, all groups of drugs could reduce the levels of inflammatory factors in the serum, among which the reduction degree of the ZLR-H group was the most obvious (Figure 5D). These results indicate that ZLR can, to some extent, prevent HFD-induced oxidative damage in NAFLD rats and slow down the transformation of NAFLD to NASH.

### 3.7. Transcriptomic Analysis Revealed That the Treatment of Stir-Roasted Deer Antler Had a Significant Impact on Fatty Acid Metabolism

**Figure 6:**
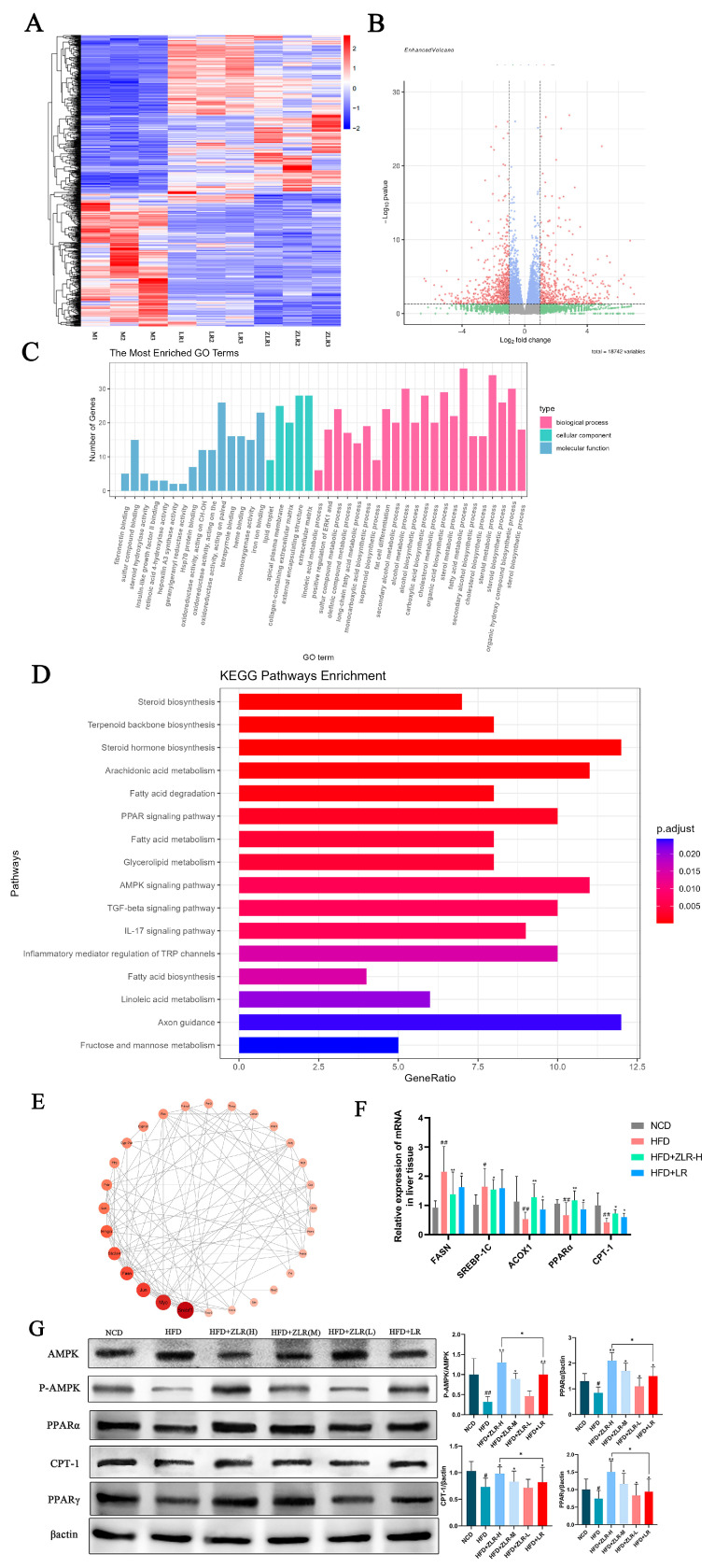
Stir-roasted activates AMPK/PPAR signaling, thereby modulating lipid metabolism. (**A**) Clustering heatmaps of the model group, deer antler group, and stir-roasted deer antler group. (**B**) Volcano map of the HFD vs. ZLR + HFD group. (**C**) GO analysis results. (**D**) EGG analysis results. (**E**) pip network mutual mapping. (**F**) The relative mRNA levels of lipid metabolism-related genes. (**G**) Levels of Various protein in each group. The data is expressed as standard deviation ± mean. n = 5. * *p* < 0.05, ** *p* < 0.01, ^#^ *p* < 0.05, ^##^ *p* < 0.01. (* indicates the comparison between the drug-treated group and the model group; # indicates the comparison between the model group and the blank control group).

To characterize the global transcriptome profile, RNA sequencing (RNA-seq) was performed using rat liver tissue samples, followed by the construction of gene expression heatmaps and volcano plots for differential expression analysis (Figure 6A,B). As shown in the figures, a total of 18,742 differentially expressed genes (DEG) were found, among which 327 genes were upregulated, 258 genes were downregulated, and the rest were meaningless. Further analysis and prediction were conducted using GO and KEGG. The biological processes, cell grouping, and molecular function annotations are shown in Figure 6C. It was found that ZLR has regulatory effects on biological processes such as lipid metabolism, steroid hormone metabolism, inflammation, fibrosis, and apoptosis. Stir-roasted deer antler velvet exerted a partial ameliorative effect on the aberrant transcriptional alterations in genes (including ALOX15 and Gstm5) involved in the aforesaid pathway. Furthermore, Kyoto Encyclopedia of Genes and Genomes (KEGG) enrichment analysis demonstrated that ZLR administration predominantly modulated the biological processes associated with fatty acid metabolism and catabolism in non-alcoholic fatty liver disease (NAFLD) model rats (Figure 6D). Construction of a protein–protein interaction network from the differentially expressed genes between model and ZLR-treated groups revealed key hub nodes, notably the transcription factors SREBF1, FASN, MYC, JUN, and CYP17A1. Subsequent functional annotation mapped these core factors to the AMPK and PPAR signaling cascades (Figure 6E). Both pathways are central regulators of cellular metabolism. The metabolic benefits of AMPK activation are mediated through complementary pathways: potentiation of CPT-1-mediated fatty acid oxidation and suppression of lipogenic gene expression. Specifically, AMPK phosphorylation enhances mitochondrial/peroxisomal β-oxidation while concurrently inhibiting SREBP-1c nuclear translocation and FASN transcription, creating a coordinated response that alleviates hepatic steatosis through reduced lipid synthesis and enhanced lipid clearance. Moreover, studies have shown that AMPK can influence the downstream PPAR pathway to further alleviate NAFLD [23,24,25]. Therefore, the mechanism of stir-roasted deer antler in the treatment of NAFLD may be regulated by the AMPK and PPAR signaling pathways.

### 3.8. The Liver Gene Expression Reconstructed by Pasted Deer Antler Participates in Lipid Metabolism Through the AMPK and PPAR Pathways

To explore the potential molecular mechanism of ZLR in the regulation of fatty acid metabolism, the relative mRNA levels of lipid synthesis promoting genes in liver tissue were detected by RT-qPCR. Including FASN, sterol regulatory element-binding protein-1c (SREBP-1c), and genes that accelerate fat breakdown including acyl-coA oxidase 1 (ACOX1), CPT-1, and PPARα. The results (Figure 6F) showed that the mRNA levels of FASN and SREBP-1c in HFD-induced NAFLD rats were significantly higher than those in the blank group, and the levels of ACOX1, PPARα, and CPT-1 were downregulated. After administration with ZLR, the mRNA levels can be improved, and ZLR has a more significant effect on the mRNA levels of ACOX1, PPARα, and FASN.

Furthermore, in order to further clarify whether ZLR alleviates HFD-induced lipid metabolism by activating AMPK and regulating the expression of downstream PPAR, the expression levels of AMPK, p-AMPK, PPAR α, CPT-1, and PPARγ in the liver were detected. As shown in Figure 6F, we found that ZLR could improve the expression of lipid metabolism-related genes in the liver of HFD-induced NAFLD rats in a dose-dependent manner. Among them, HFD can significantly inhibit the protein levels of AMPK and phosphorylated AMPK, while treatment with ZLR can significantly improve the expressions of AMPK and P-AMPK. Meanwhile, ZLR can increase the expression of PPARα and CPT-1 and inhibit the protein level of PPARγ. In conclusion, the results suggest that ZLR may regulate lipid metabolism through the AMPK and PPAR pathways, indicating that both could be potential therapeutic targets for ZLR in the treatment of NALFD.

The above results indicate that ZLR can further alleviate the deterioration of NAFLD by improving the fat metabolism disorder in NAFLD and reducing the accumulation of lipids in the liver.

### 3.9. Stir-Roasted Deer Antler Inhibits the Lipid Accumulation and Oxidative Stress Levels of AML12 Cells Induced by OA

A model of excessive fat accumulation in mouse hepatocyte lines (AML12) was established by incubating with sodium oleate (OA) in vitro for 24 h [9,26,27].

**Figure 7:**
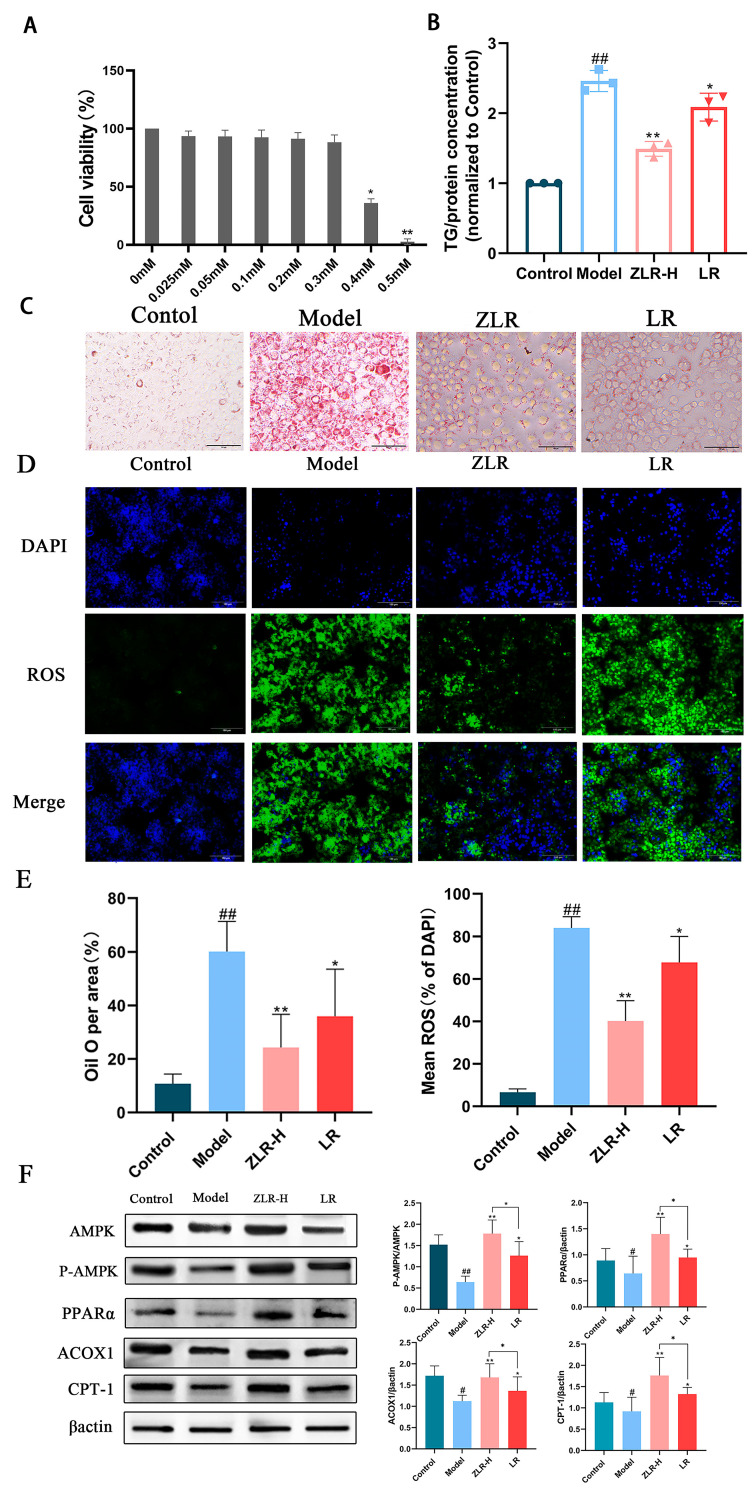
Effects of stir-roasting on lipid metabolism and oxidative stress in AML12 cells induced by sodium oleate. (**A**) Cytotoxicity of sodium oleate. (**B**) TG content of the cell supernatant. (**C**) Cell oil red O staining. (**D**) ROTH staining. (**E**) Determination of quantitative levels of oil red O and ROS. (**F**) Expression levels of proteins related to lipid metabolism. The data is expressed as standard deviation ± mean. The n of all groups is 8. * *p* < 0.05, ** *p* < 0.01, ^#^ *p* < 0.05, ^##^ *p* < 0.01. (* indicates the comparison between the drug-treated group and the model group; # indicates the comparison between the model group and the blank control group).

According to the results of the MTT assay (Figure 7A), OA at concentrations below 0.3 mM exerted minimal cytotoxicity on AML12 cells, allowing the cells to grow normally without undergoing apoptosis. Therefore, the concentration of 0.3 mM was selected for subsequent experiments. Results of the TG content detection in AML12 cells (Figure 7B) showed that the drug-containing serum from the ZLR group of rats could also reduce TG synthesis in hepatocytes in vitro. Subsequently, Oil Red O staining was performed on the cells. As indicated in Figure 7C, after treatment, the sera from both the ZLR and LR groups could reduce lipid droplet accumulation in hepatocytes to varying degrees. However, the drug-containing serum from the ZLR group exhibited a significantly superior effect compared with that from the LR group, and could markedly decrease the accumulation of intracellular lipid droplets. In addition, NAFLD can progress to NASH over time. Mechanistically, NF-κB activation by liver-derived inflammatory factors (IL-6, TNF-α) serves as a critical upstream signal for NLRP3 inflammasome activation in this process. Reactive oxygen species (ROS) are common upstream signaling molecules of NLRP3 inflammasome activation [28], and their level changes can effectively reflect the severity of NAFLD. Therefore, we further detected the production levels of ROS in each group through immunofluorescence kits. The results are shown in Figure 7D. Compared with the blank group, the model group produced significantly more reactive oxygen species (ROS), and the model group produced the most ROS. Both the ZLR and LR groups could reduce the production of ROS, and the ZLR group could more significantly lower the ROS content in the cells (Figure 7E is the quantification plot of Oil Red O and ROS staining).

### 3.10. The Effect of Stir-Roasted Deer Antler on Lipid Metabolism-Related Proteins in OA-Induced AML12 Cells

In order to further evaluate the effect of ZLR on lipid metabolism-related proteins, the expressions of AMPK, P-AMPK, PPARα, ACOX1, and CPT-1 were detected. The results are shown in Figure 7F. The LR group and the ZLR group can promote the phosphorylation process of AMPK and increase the expression of PPARα, ACOX1, and CPT-1, among which the effect of the ZLR group is particularly significant. The above results indicate that ZLR can effectively inhibit lipid synthesis and enhance lipid metabolism by regulating lipid metabolism-related proteins.

### 3.11. Study on the Effect of ZLR on AMPK Knockdown Cells Induced by OA

**Figure 8:**
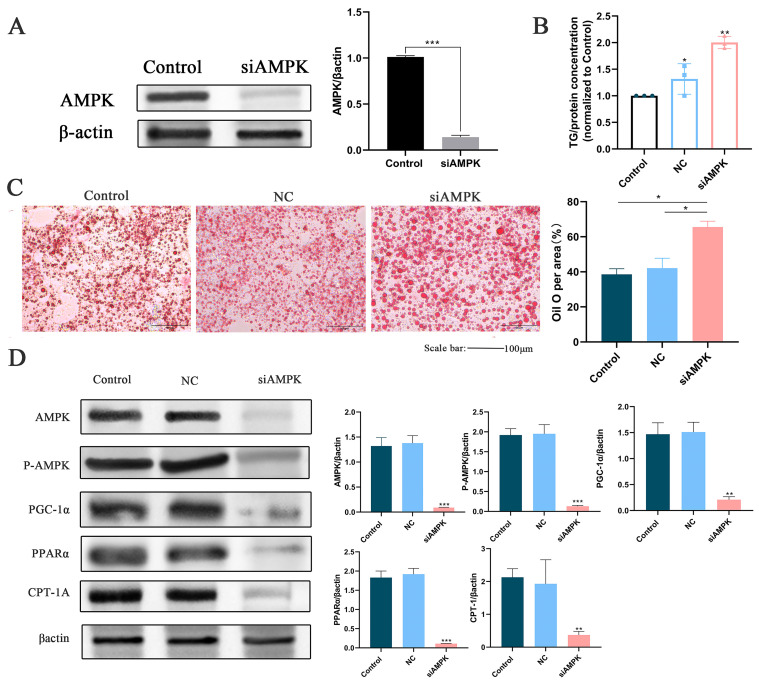
Effects of AMPK knockout on PPARα and genes related to lipid metabolism. (**A**) Verification of the silencing effect of the APK gene. (**B**) Determination of the relative content of TG in cell supernatant. (**C**) Oil red O staining. (**D**) The effect of AMPK knockout on protein levels. The data is expressed as standard deviation ± mean. The n of all groups is 3. * *p* < 0.05, ** *p* < 0.01, *** *p* < 0.001.

In this experiment, AML12 cells were further transfected with siRNA interference sequences to silence the AMPK gene, in order to explore whether ZLR’s regulation of OA-induced anti-NAFLD depends on the AMPK/PPAR signaling. Figure 8A shows the efficiency of AMPK gene silencing. Subsequently, the TG content in the cell supernatant was determined (Figure 8B), and Oil Red O staining was performed (Figure 8C). The results indicated that after AMPK knockdown, the inhibitory effect of ZLR on TG content was attenuated, and its ability to suppress lipid droplet formation was significantly weakened. Figure 8D illustrates the changes in the expression levels of lipid metabolism-related genes following AMPK silencing. It can be observed that, after AMPK silencing, the expression of downstream lipid metabolism-related proteins, such as PPARα and CPT-1A, was correspondingly downregulated.

## 4. Discussion

The rising global incidence of NAFLD presents a pressing global health challenge, drawing considerable scientific and clinical interest due to its substantial disease burden. The development of effective treatment strategies is extremely urgent. Deer antler, as a classic traditional Chinese medicine, has a wide range of therapeutic effects. In terms of liver protection and fat metabolism, Tong Sun et al. confirmed that deer antler polypeptides can improve the degeneration of fat cells through the AMPK signaling pathway [29]. As pivotal regulators of cellular metabolism, the AMPK and PPAR signaling pathways represent promising therapeutic targets for the prevention and treatment of NAFLD [30]. Hepatic steatosis usually results from excessive calorie intake and the accumulation of TGs. Among them, the free fatty acids esterified into liver TGs can either come from the lipolysis of adipose tissue or be produced by de novo fat synthesis in the liver [31]. Activation of AMPK can inhibit the maturation and transcriptional activity of SREBP-1c, thereby reducing lipid regeneration [32]; meanwhile, AMPK can also upregulate the expression of PPARα and its target genes, promote fatty acid oxidation (FAO), and further reduce lipid deposition in the liver. As a nuclear hormone receptor, PPARα plays a core role in the catabolism of fatty acids. Its activation can effectively promote the β-oxidation process in mitochondria and peroxisomes [29,31,33]. From the perspective of overall energy metabolism, the activation of AMPK, as a cellular energy sensor, not only inhibits lipid synthesis that consumes energy but also promotes fatty acid oxidation that generates energy, which is conducive to restoring the energy homeostasis of liver cells.

Based on the above background, this study first determined the protein components in ZLR through proteomics and found that compared with LR, ZLR contains 1789 kinds of proteins such as DHX57, protein-arginine deiminase, BTB domain-containing protein, Histone acetyltransferase, etc. Among them, the protein components related to dairy products have significantly increased. The top ten signals of the protein and polypeptide of the stir-roasted deer antler are shown in Tables S1 and S2. Among them, proteins closely related to NAFLD include BTB domain-containing proteins (BTB domain proteins) and Histone acetyltransferase (histone acetyltransferase). On the one hand, BTB domain proteins are negatively correlated with NAFLD [34,35]. Their high expression can upregulate the expression of fatty acid oxidation-related genes (such as CPT1A and ACOX1), accelerate the β-FFA in liver cells, break the imbalance where fatty acid synthesis exceeds consumption, and reduce lipid deposition in liver cells. Secondly, the BTB domain protein can stimulate the expression of mesh protein heavy chains (CLTC), physically bind to NLRP3 protein, and prevent its assembly into functional inflammatomes, thereby reducing the release of pro-inflammatory factors such as IL-1β and alleviating liver inflammatory infiltration. On the other hand, Histone acetyltransferase can acetylate HNF4α (a key factor involved in liver metabolic regulation), and the acetylation state of HNF4α will affect its transcriptional activation of lipid metabolism-related genes [36], further maintaining the regulatory function of metabolism. It can help maintain the balance of lipid metabolism in the liver, thereby exerting a certain inhibitory and alleviating effect on NAFLD.

The protein functions corresponding to the top ten peptides that cover the lipid metabolism regulation, antioxidation and anti-inflammation, optimization of energy metabolism, and maintenance of microenvironmental homeostasis in the pathological process of NAFLD, and can synergistically regulate multiple targets of NAFLD. Among them, YAEAETLYK is a homologous protein of apolipoprotein B-100 (APOB). It can mediate the binding of triglycerides and lipoproteins in the liver, promote the transport of lipids to peripheral tissues, reduce fat accumulation in hepatocytes, lower the synthesis disorder of very low-density lipoproteins in the liver, and reduce TG and LDL-C. The parent proteins of RHPYFYAPELLYYANK, VMQQNLVYYQYHR, and LYGVYCFR are HSP70, GST homologous protein and SOD homologous protein, which can eliminate ROS, reduce MDA production, protect liver cells, and reduce liver cell necrosis and apoptosis caused by oxidative stress. QHFCGGSLIAPEWVLTAK and GLLEELKR are homologous proteins of MDH2 and PGC-1α. Both can enhance mitochondrial function, jointly promote the β -oxidation of fatty acids, improve insulin resistance, regulate the balance of glycolipid metabolism, reduce hepatic gluconeogenesis, and alleviate the vicious cycle of fat accumulation caused by metabolic disorders. EDAGGMIQR, as an ACTB homologous protein, can stabilize the cytoskeleton of hepatocytes and prevent the structural rupture of cells due to lipid toxicity, and serum albumin can bind to free fatty acids, reducing their direct toxicity to hepatocytes, maintaining microenvironmental homeostasis, and regulating local osmotic pressure and nutrient supply in the liver.

Meanwhile, as the core excipient for processing ZLR, ghee is composed of vitamins, fatty acids, lipids, proteins, minerals, and other components. Its role is not merely a “carrier” for protein components. Numerous studies have confirmed that ghee is rich in a variety of components with anti-NAFLD activities [37,38,39], including fatty acids (conjugated linoleic acid (CLA), α-linolenic acid, etc.), lipids (phosphatidylcholine, sphingomyelin, etc.) that can improve hepatocyte membrane stability and promote lipid transport, bioactive small molecules (vitamin E, β-carotene, etc.), and proteins (α-casein, β-casein, etc.). In previous relevant studies, these components have been shown to reduce NAFLD risk factors such as obesity and insulin resistance by improving lipid metabolism, inhibiting lipid synthesis, and exerting antioxidant effects, thereby decreasing the incidence of the disease. Combined with the proteomics results of this study and the core findings under the current sample size, it is speculated that the proteins in ZLR can form a synergistic anti-NAFLD effect with various components in ghee through “target synergy” and “pathway complementarity.”

Subsequently, we used an HFD-induced rat model of NAFLD to systematically evaluate the therapeutic potential and mechanism of action of ZLR against this disease. Experimental results showed that compared with the NCD group, after 12 weeks of HFD feeding, the body mass, liver weight, and degree of hepatic steatosis in the model group were significantly increased. Intervention with ZLR powder or common deer antler (LR) powder for 4 weeks significantly reduced HFD-induced body mass gain, liver weight, and hepatic lipid accumulation in NAFLD rats without affecting food intake. Notably, ZLR also significantly improved HFD-induced hyperlipidemia, impaired glucose tolerance, and insulin resistance. Pathological evaluation was performed with an equivalent dose (1.35 g/kg/d) of LR as the positive control, and it was found that the therapeutic effect of ZLR was superior to that of LR. Furthermore, the detection of oxidative stress-related indicators (lipid oxidation end product MDA, antioxidant enzyme SOD, and reduced GSH) revealed that ZLR could effectively ameliorate the oxidative stress state associated with NAFLD. These results indicate that ZLR can effectively alleviate the pathological progression of NAFLD.

It should be noted that this study only used 5 rats per group, which indeed has the limitation of a small sample size. To a certain extent, this may increase the risk of individual fluctuations in metabolic indicators and lead to insufficient statistical reliability of some secondary indicators. To mitigate this limitation as much as possible, we enhanced the credibility of the results through two aspects: statistical design and experimental quality control. First, post hoc power analysis was performed using G*Power 3.1 software. For core outcome indicators such as hepatic TG content and BTB domain-containing protein expression, the calculated effect size (Cohen’s d) was 1.23, and the statistical power (1-β) was 0.82 under the conditions of α = 0.05 and n = 5. This meets the statistical test requirements for core indicators in metabolic disease research and can effectively detect true intergroup differences. Second, individual differences were strictly controlled during the experiment: rats were randomly assigned to groups using a random number table; pathological section evaluation and indicator detection were conducted in a single-blind manner to avoid subjective bias; the formula of HFD, feeding environment, and intervention dose were standardized; and all indicators were measured in triplicate, and no outliers were excluded after Grubbs test, which minimized the interference of random errors on the results. Notably, as an animal-derived processed product, ZLR is associated with allergen-related concerns of the animal-derived components and contamination residue risks. For its future clinical translation, it is necessary to clarify the applicable population scope and ensure compliance with the Pharmacopoeia of the People’s Republic of China as well as food and drug safety standards. In addition, regarding long-term medication safety, this study was a short-term intervention experiment. Therefore, long-term animal toxicity studies are required to evaluate the potential impacts of ZLR on hepatic and renal functions as well as the hematological system, so as to provide data support for its long-term clinical application.

Based on the verification of therapeutic effects, this study delved deeply into the mechanism of action of stir-roasted deer antler. Given that the dose group of 1.35 g/kg/d (HFD + ZLR-H) had the best therapeutic effect, we selected it as the representative group for transcriptome analysis. stir-roasted deer antler treatment effectively reversed the HFD-induced upregulation of key lipogenic genes (FASN and SREBP-1c) in the liver, as demonstrated by molecular mechanism studies. Conversely, the transcriptional suppression of pivotal lipid-catabolizing genes (ACOX1, CPT-1, PPARα) induced by HFD was robustly reversed by ZLR treatment, which restored their expression to promote fatty acid β-oxidation. Peroxisome proliferator-activated receptor alpha is a core transcription factor regulating the β-oxidation of fatty acids and is crucial for limiting lipid accumulation in the liver [30]. CPT-1, located downstream of PPARα, is a key factor in regulating fatty acid β-oxidation and reducing lipid deposition in the liver [24,40]. The Western blot results further confirmed that stir-roasted deer antler significantly increased the protein expression levels of PPARα and CPT-1 in the liver of HFD model rats. In conclusion, stir-roasted deer antler may improve liver steatosis by inhibiting fat production while promoting lipid decomposition and fatty acid oxidation. The AMPK signaling pathway plays a core role in regulating energy balance and maintaining the homeostasis of glycolipid metabolism, and it is a potential target for treating metabolic diseases. This study found that compared with the NCD group, the level of phosphorylated AMPK (p-AMPK) protein in the liver of rats in the HFD group decreased, while the level of PPARγ protein increased. The treatment of stir-roasted deer antler significantly increased the expression of p-AMPK and inhibited the expression of PPARγ, indicating that its mechanism of action involves activating the AMPK signaling pathway and its downstream PPAR pathway.

To further verify the conclusions of the in vivo experiments, this study conducted in vitro cytological validation using drug-containing serum. The results demonstrated that the drug-containing serum of stir-roasted deer antler with ghee could significantly inhibit OA-induced hepatic cell lipid droplet accumulation and ROS production and exert a prominent regulatory effect on the expression levels of key lipid metabolism-related proteins, including PPARα, ACOX1, and CPT-1, which was in good agreement with the findings of the in vivo experiments. To address the inherent limitations of serum pharmacology, such as complex drug components, difficulty in accurately quantifying effective concentrations, and susceptibility to interference from serum matrix, a series of optimization measures were implemented during the preparation of drug-containing serum in this study: strictly selecting standardized experimental animals, precisely controlling blood collection time points, and uniformly regulating the addition ratio of drug-containing serum in the cell culture system. These measures served to minimize experimental errors to the greatest extent and improve the reliability and specificity of the in vitro experimental results.

Finally, to investigate the mechanism underlying the effects of ZLR, we performed a functional validation experiment using siRNA-mediated AMPK gene silencing in an OA-induced AML12 cell model. The experimental results showed that after stable knockdown of AMPK expression, the inhibitory effects of ZLR on OA-induced hepatocyte TG accumulation and lipid droplet formation were significantly attenuated, while its regulatory effects on the expression of downstream lipid metabolism-related proteins (e.g., PPARα and CPT-1A) were also diminished concomitantly. These findings directly confirm that the anti-NAFLD effects of ZLR are dependent on the mediation of the AMPK/PPAR signaling pathway, which fills the gap in the previous mechanistic speculations based solely on transcriptome enrichment analysis, establishes a definitive causal link between ZLR intervention, AMPK/PPAR pathway activation, and the improvement in hepatocyte lipid homeostasis, and provides robust and reliable experimental evidence for the direct regulatory role of ZLR in this signaling pathway.

## 5. Conclusions

In summary, this study not only provides scientific evidence for the modern application of the traditional Chinese medicinal preparation of fried deer antler, confirming its potential in NAFLD treatment, but also establishes a novel approach for clinical NAFLD therapy by identifying the AMPK/PPAR signaling pathway as a core mechanism. This approach involves “exploring effective interventions from traditional Chinese medicine and seeking precise regulatory targets within metabolic pathways.” Furthermore, it lays an experimental foundation for the subsequent development of NAFLD therapeutic drugs or auxiliary intervention protocols based on fried deer antler and identifying precise regulatory targets within metabolic pathways. It also lays the experimental foundation for subsequent development of NAFLD therapeutic drugs or adjunctive intervention protocols based on suzhi deer antler.

## Figures and Tables

**Table 1:** Antibody used for WB.

Antibody	Manufacturer
ACOX1	BYabscience; BYab-17982
PPARα	Wanleibio
PPARγ	BYabscience; BYab-17828
CPT-1	BYabscience; BYab-08231
p-AMPK	Wanleibio
AMPK	Wanleibio
β-Actin	Cellorlab; LF212

**Table 2:** Primers used for RT-qPCR.

Gene	Sequences 5′-3′ Forward	Sequences 5′-3′ Reverse
*β-actin*	CATGTACGTTGCTATCCAGGC	CTCCTTAATGTCACGCACGAT
*PPAR* *α*	AGAGCCCCATCTGTCCTCTC	ACTGGTAGTCTGCAAAACCAAA
*CPT-1*	TATGGTCAAGGTCTTCTCGGGTCG	AGTGCTGTCATGCGTTGGAAGTCTC
*ACOX1*	TAACTTCCTCACTCGAAGCCA	AGTTCCATGACCCATCTCTGTC
*FASN*	GGAGGTGGTGATAGCCGGTAT	TGGGTAATCCATAGAGCCCAG
*Srebp-1c*	GTGAGGCGGCTCTGGAACAGAC	ATAGGGGGCGTCAAACAGGCC

**Table 3:** AST and ALT levels in serum.

Group	n	AST	ALT
Normal group	5	23.32 ± 5.86	46.28 ± 8.62
Model group	25	43.25 ± 13.86	96.23 ± 33.28

## Data Availability

Data is contained within the article or Supplementary Materials. The original contributions presented in this study are included in the article/Supplementary Materials. Further inquiries can be directed to the corresponding authors.

## Supplementary Materials

The following supporting information can be downloaded at: https://www.mdpi.com/article/10.3390/nu18030401/s1, Table S1. Top 10 Differentially Expressed Proteins in Ghee-Stir-Fried and Roasted Deer Antler; Table S2. Top 10 Ranking of Peptide Differences in Ghee-Stir-Fried and Roasted Deer Antler.

## Abbreviations

ZLR	Stir-roasted deer velvet antler with ghee
NAFLD	non-alcoholic fatty liver disease
HFD	high-fat diet
NCD	normal chow diet
FFA	free fatty acids
LR	deer antler velvet
TG	triglyceride
TC	total cholesterol
MDA	malondialdehyde
SOD	superoxide dismutase
GSH	glutathione
MTT	methyl thiazolyl tetrazolium
ALT	Alanine Aminotransferase
AST	aspartate aminotransferase
IPGTT	intraperitoneal glucose tolerance test
IPITT	intraperitoneal insulin tolerance tests
CPT-1	carnitine palmitoyltransferase 1
KEGG	Kyoto Encyclopedia of Genes and sterol regulatory
FANS	fatty acid synthase
SREBP-1c	sterol regulatory element-binding protein 1c
ACOX1	Acyl-CoA Oxidase 1
OA	oleic acid
ROS	reactive oxygen species

## References

[1] Gofton C., Upendran Y., Zheng M.-H., George J. (2023). MAFLD: How is it different from NAFLD?. Clin. Mol. Hepatol..

[2] Bugianesi E., Petta S. (2022). NAFLD/NASH. J. Hepatol..

[3] Dong Y., Liu L., Shan X., Tang J., Xia B., Cheng X., Chen Y., Tao W. (2018). Pilose antler peptide attenuates LPS-induced inflammatory reaction. Int. J. Biol. Macromol..

[4] Xu R., Pan J., Zhou W., Ji G., Dang Y. (2022). Recent advances in lean NAFLD. Biomed. Pharmacother..

[5] Rojano A., Sena E., Manzano-Nuñez R., Pericàs J.M., Ciudin A. (2023). NAFLD as the metabolic hallmark of obesity. Intern. Emerg. Med..

[6] Abasubong K.P., Jiang G.-Z., Guo H.-X., Wang X., Huang Y.-y., Li X.-F., Yan-zou D., Liu W.-b., Desouky H.E. (2024). Effects of a high-fat and high-carbohydrate diet on appetite regulation and central AMPK in the hypothalamus of blunt snout bream (Megalobrama amblycephala). J. Anim. Physiol. Anim. Nutr..

[7] López M., Nogueiras R., Tena-Sempere M., Diéguez C. (2016). Hypothalamic AMPK: A canonical regulator of whole-body energy balance. Nat. Rev. Endocrinol..

[8] Świderska M., Maciejczyk M., Zalewska A., Pogorzelska J., Flisiak R., Chabowski A. (2019). Oxidative stress biomarkers in the serum and plasma of patients with non-alcoholic fatty liver disease (NAFLD). Can plasma AGE be a marker of NAFLD? Oxidative stress biomarkers in NAFLD patients. Free Radic. Res..

[9] Lin Q., Cai G., Liu Z., Li J., Li J., Wintergerst K.A., Epstein P.N., Cai L., Li Y., Tan Y. (2020). 1831-P: Activating AMPK Mediates FGF1 Protection from NAFLD in Diabetic Mice. Diabetes.

[10] Chen J., Montagner A., Tan N.S., Wahli W. (2018). Insights into the Role of PPARβ/δ in NAFLD. Int. J. Mol. Sci..

[11] Lijuan C., Wen L., Yanli F., Yuhe L., Wenjian X., Suihua R., Ning L., Miaomiao Z., Jiayi H., Yanfen C. (2024). Shugan Jiangzhi Decoction Alleviates Nonalcoholic Fatty Liver Disease (NAFLD) via Regulating AMPK/PPAR Signaling Pathway. Lett. Drug Des. Discov..

[12] Dai W., Hou Q., Ye J. (2025). Rhein alleviates hepatic steatosis in NAFLD mice by activating the AMPK/ACC/SREBP1 pathway to enhance lipid metabolism. Mol. Med..

[13] Garcia D., Hellberg K., Chaix A., Wallace M., Herzig S., Badur M.G., Lin T., Shokhirev M.N., Pinto A.F.M., Ross D.S. (2019). Genetic Liver-Specific AMPK Activation Protects against Diet-Induced Obesity and NAFLD. Cell Rep..

[14] Li L., Yang F., Jia R., Yan P., Ma L. (2020). Velvet antler polypeptide prevents the disruption of hepatic tight junctions via inhibiting oxidative stress in cholestatic mice and liver cell lines. Food Funct..

[15] Sun H., Xiao D., Liu W., Li X., Lin Z., Li Y., Ding Y. (2024). Well-known polypeptides of deer antler velvet with key actives: Modern pharmacological advances. Naunyn-Schmiedeberg’s Arch. Pharmacol..

[16] Chen S., Li Y., Yang Y., Zhao S., Shi H., Yang C., Wu M., Zhang A. (2024). Comparison of the composition, immunological activity and anti-fatigue effects of different parts in sika deer antler. Front. Pharmacol..

[17] Ding Y., Wang Y., Jeon B.-T., Moon S.-H., Lee S.-H. (2017). Enzymatic hydrolysate from velvet antler suppresses adipogenesis in 3T3-L1 cells and attenuates obesity in high-fat diet-fed mice. EXCLI J..

[18] Xu L., Yan L., Tao W. (2018). Pilose antler peptide attenuates high-fat-diet-induced liver injury. Toxicol. Mech. Methods.

[19] Piccinin E., Moschetta A. (2016). Hepatic-specific PPARα-FGF21 action in NAFLD. Gut.

[20] Khan R.S., Bril F., Cusi K., Newsome P.N. (2019). Modulation of Insulin Resistance in Nonalcoholic Fatty Liver Disease. Hepatology.

[21] Zhao H., Zhai B.-W., Zhang M.-Y., Huang H., Zhu H.-L., Yang H., Ni H.-Y., Fu Y.-J. (2024). Phlorizin from Lithocarpus litseifolius [Hance] Chun ameliorates FFA-induced insulin resistance by regulating AMPK/PI3K/AKT signaling pathway. Phytomedicine.

[22] Lei Y., Wang L., Song L., Han J., Ma H., Luo H., Ma Y., Han D. (2025). Tiaogan Jiejiu Tongluo formula alleviates hepatic steatosis in NAFLD mice by regulating AMPK signaling pathway. J. Pharm. Pharmacol..

[23] You L., Wang T., Li W., Zhang J., Zheng C., Zheng Y., Li S., Shang Z., Lin J., Wang F. (2024). Xiaozhi formula attenuates non-alcoholic fatty liver disease by regulating lipid metabolism via activation of AMPK and PPAR pathways. J. Ethnopharmacol..

[24] Diniz T.A., de Lima Junior E.A., Teixeira A.A., Biondo L.A., da Rocha L.A.F., Valadão I.C., Silveira L.S., Cabral-Santos C., de Souza C.O., Rosa Neto J.C. (2021). Aerobic training improves NAFLD markers and insulin resistance through AMPK-PPAR-α signaling in obese mice. Life Sci..

[25] Wu L., Liu C., Chang D.-Y., Zhan R., Zhao M., Man Lam S., Shui G., Zhao M.-H., Zheng L., Chen M. (2021). The Attenuation of Diabetic Nephropathy by Annexin A1 via Regulation of Lipid Metabolism Through the AMPK/PPARα/CPT1b Pathway. Diabetes.

[26] Norikura T., Mukai Y., Fujita S., Mikame K., Funaoka M., Sato S. (2010). Lignophenols Decrease Oleate-Induced Apolipoprotein-B Secretion in HepG2 Cells. Basic Clin. Pharmacol. Toxicol..

[27] Bort A., Sánchez B.G., Mateos-Gómez P.A., Díaz-Laviada I., Rodríguez-Henche N. (2019). Capsaicin Targets Lipogenesis in HepG2 Cells Through AMPK Activation, AKT Inhibition and PPARs Regulation. Int. J. Mol. Sci..

[28] Linghu L., Zong W., Liao Y., Chen Q., Meng F., Wang G., Liao Z., Lan X., Chen M. (2023). Herpetrione, a New Type of PPARα Ligand as a Therapeutic Strategy Against Nonalcoholic Steatohepatitis. Research.

[29] Sun T., Hao Z., Meng F., Li X., Wang Y., Zhu H., Li Y., Ding Y. (2025). The Effects of Sika Deer Antler Peptides on 3T3-L1 Preadipocytes and C57BL/6 Mice via Activating AMPK Signaling and Gut Microbiota. Molecules.

[30] Tu Z., Moss-Pierce T., Ford P., Jiang T.A. (2013). Rosemary (*Rosmarinus officinalis* L.) Extract Regulates Glucose and Lipid Metabolism by Activating AMPK and PPAR Pathways in HepG2 Cells. J. Agric. Food Chem..

[31] Liu X., Yang Q., Li H., Lan X., Kan M., Lin J., Wang J., Zhang Z., Ming S., Li Z. (2021). The anti-aging effect of velvet antler polypeptide is dependent on modulation of the gut microbiota and regulation of the PPARα/APOE4 pathway. J. Integr. Neurosci..

[32] Wang M., Wang B., Wang S., Lu H., Wu H., Ding M., Ying L., Mao Y., Li Y. (2021). Effect of Quercetin on Lipids Metabolism Through Modulating the Gut Microbial and AMPK/PPAR Signaling Pathway in Broilers. Front. Cell Dev. Biol..

[33] Ke W., Huang J., Zhong Y., Shi Y., Yan F., Huang D., Wu Y., Zheng H., Weng Z. (2023). Hydroxypropyl-beta-Cyclodextrin embedded resveratrol regulates gut microbiota to prevent NAFLD via activating AMPK signaling pathway. Food Biosci..

[34] Zhang W., Tian Z., Qi X., Chen P., Yang Q., Guan Q., Ye J., Yu C. (2023). Switching from high-fat diet to normal diet ameliorate BTB integrity and improve fertility potential in obese male mice. Sci. Rep..

[35] Zhou J.-p., Ren Y.-d., Xu Q.-y., Song Y., Zhou F., Chen M.-Y., Liu J.-j., Chen L.-G., Pan J.-S. (2020). Obesity-Induced Upregulation of ZBTB7A Promotes Lipid Accumulation through SREBP1. BioMed Res. Int..

[36] Huh J.Y., Saltiel A.R. (2021). Roles of IκB kinases and TANK-binding kinase 1 in hepatic lipid metabolism and nonalcoholic fatty liver disease. Exp. Mol. Med..

[37] Jing B., Chen W., Wang M., Mao X., Chen J., Yu X. (2019). Traditional Tibetan Ghee: Physicochemical Characteristics and Fatty Acid Composition. J. Oleo Sci..

[38] Ulambayar N.-E., Smanalieva J., Hellwig A., Iskakova J., Choijilsuren N., Kalemshariv B., Vankhuu E. (2024). Nutritional composition of ghee of various animal origins produced in some silk road countries. J. Food Compos. Anal..

[39] Kataria D., Singh G. (2024). Health benefits of ghee: Review of Ayurveda and modern science perspectives. J. Ayurveda Integr. Med..

[40] Guthrie G. (2022). Parenteral Nutrition Associated Hepatic Steatosis and NAFLD Intersect at AMPK. Cell. Mol. Gastroenterol. Hepatol..

