# Supplementary material for: Research on the Therapeutic Effect and Mechanism of Stir-Roasted Deer Velvet Antler with Ghee on Non-Alcoholic Fatty Liver Disease
Source: Nutrients. 2026 Jan 26;18(3):401. doi: 10.3390/nu18030401 (PMC12899000; doi:10.3390/nu18030401)
Supplement: Supplementary file 1 [file nutrients-18-00401-s001.zip › nutrients-4073821-supplementary.pdf]

nutrients

MDPI

Supplementary Materials

Table S1. Top 10 Differentially Expressed Proteins in Ghee-Stir-Fried and Roasted Deer Antler.

Rank	Protein Name	Gene	ZLR
1	DHX57	Celaphus_00005682	268961934.4
2	protein-arginine deiminase	Celaphus_00014682	185868660.6
3	Protein transport protein Sec61 subunit gamma	Celaphus_00010188	160606000
4	COL9A2	Celaphus_00002694	159180000
5	BTB domain-containing protein	Celaphus_00003579	137171000
6	Uncharacterized protein	Celaphus_00002496	98911856.99
7	Histone acetyltransferase	Celaphus_00019372	90455989.77
8	RPL26L1	Celaphus_00017888	87147399.42
9	NIFK	Celaphus_00015964	85971646.22
10	Polycomb protein EED	Celaphus_00013599	74333300

Table S2. Top 10 Ranking of Peptide Differences in Ghee-Stir-Fried and Roasted Deer Antler.

Rank	Peptide Sequence	Gene	ZLR	Corresponding protein name
1	YAEAETLYK	Celaphus_00014325	1156510000	Apolipoprotein B-100 (APOB) homolog
2	RHPYFYAPELLYYANK	Celaphus_00017423	1106303502	Heat shock protein 70 (HSP70) homolog
3	VMQQNLVYYQYHR	Celaphus_00010221	606720199	Glutathione S-transferase (GST) homolog
4	QHFCGGSLIAPEWVLTAK	Celaphus_00019151	550486000	Mitochondrial malate dehydrogenase (MDH2) homolog
5	EDAGGMIQR	Celaphus_00000247	472404000	Actin beta (ACTB) homolog
6	GLLEELKR	Celaphus_00001845	351067000	Peroxisome proliferator-activated receptor gamma coactivator 1-alpha (PGC-1α) homolog
7	VPTPNVSVVDLTCR		328977231.6	Serum albumin (Cervidae) homolog
8	LYGVYCFR	Celaphus_00003852	294105000	Superoxide dismutase (SOD) homolog
9	IVSNASCTTNCLAPLAK		289843451	Serum albumin (Cervidae) homolog
10	CCTESLVNR	Celaphus_00017423	284412000	Heat shock protein 70 (HSP70) homolog
